# Traditional Medicine to Modern Pharmacogenomics: Ayurveda *Prakriti* Type and CYP2C19 Gene Polymorphism Associated with the Metabolic Variability

**DOI:** 10.1093/ecam/nep206

**Published:** 2011-06-08

**Authors:** Yogita Ghodke, Kalpana Joshi, Bhushan Patwardhan

**Affiliations:** ^1^Bioprospecting Laboratory, Interdisciplinary School of Health Sciences, University of Pune, India; ^2^Department of Biotechnology, Sinhagad College of Engineering, Pune 411041, India

## Abstract

Traditional Indian medicine—Ayurveda—classifies the human population into three major constituents or *Prakriti* known as *Vata, Pitta* and *Kapha* types. Earlier, we have demonstrated a proof of concept to support genetic basis for *Prakriti*. The descriptions in Ayurveda indicate that individuals with *Pitta Prakriti* are fast metabolizers while those of *Kapha Prakriti* are slow metabolizers. We hypothesized that different *Prakriti* may have different drug metabolism rates associated with drug metabolizing enzyme (DME) polymorphism. We did *CYP2C19* (Phase I DME) genotyping in 132 unrelated healthy subjects of either sex by polymerase chain reaction-restriction fragment length polymorphism (PCR-RFLP) technique. We observed significant association between *CYP2C19* genotype and major classes of *Prakriti* types. The extensive metabolizer (EM) genotype (*∗1/∗1, ∗1/∗2, ∗1/∗3*) was found to be predominant in *Pitta Prakriti* (91%). Genotype (*∗1/∗3*) specific for EM group was present only in *Pitta Prakriti.* Poor metabolizer (PM) genotype (*∗2/∗2, ∗2/∗3, ∗3/∗3*) was highest (31%) in *Kapha Prakriti* when compared with *Vata* (12%) and *Pitta Prakriti* (9%). Genotype (*∗2/∗3*) which is typical for PM group was significant in *Kapha Prakriti* (odds ratio = 3.5, *P* =  .008). We observed interesting correlations between *CYP2C19* genotypes and *Prakriti* with fast and slow metabolism being one of the major distinguishing and differentiating characteristics. These observations are likely to have significant impact on phenotype-genotype correlation, drug discovery, pharmacogenomics and personalized medicine.

## 1. Introduction

Ayurveda remains one of the most ancient and yet living traditions documented and practiced widely in India [1]. It has a time-honored philosophical and experiential basis. The core concept of health and disease in Ayurveda is built around the uniqueness of an individual [2]. Ayurveda uses a 3-fold classification known as *tridosha* theory that identifies principles of motion (*Vata*), metabolism (*Pitta*) and structure (*Kapha*) as discrete phenotypic groupings [3]. According to Ayurveda, the individual constitution or *Prakriti* classification is based on differences in physical, physiological and psychological characteristics and is independent of racial, ethnic or geographical considerations. The importance of such individual variations in health and disease is an important basic principle rightly described hundreds of years ago as “every individual is different from another and hence should be considered as a different entity; as many variations are there in the Universe, all are seen in the human being" [4–6]. The doshas exhibit more easily recognizable phenotypes. Evidence-based research in Ayurveda is receiving larger acceptance in India and abroad [7–9].

According to Ayurveda, *Prakriti* of an individual is determined at the time of conception and remains unaltered during the lifetime. *Prakriti*-specific treatment including medicine, diet and lifestyle is a distinctive feature of Ayurveda [10]. We hypothesized that *Prakriti* has a genetic connotation that can provide a tool for classifying human population based on broad phenotype clusters. We hypothesize that the human phenome based on Ayurveda can provide a genetic basis for the three major constitutions or *Prakriti.* Earlier, in a pilot study, we evaluated 76 subjects both for their *Prakriti* and HLA DRB1 typing. We observed a significant correlation between certain HLA types and *Prakriti* types [11]. For better validation, the homologous relation of *Vata* (V), *Pitta* (P) and *Kapha* (K) to human genetic structure requires further study [12].

Three major constitution types as *Vata, Pitta* and *Kapha Prakriti* have unique putative metabolic activities. *Kapha* is slow, *Pitta* is fast, while *Vata* is considered to have variable metabolism. We hypothesize that this may relate to drug metabolism and genetic polymorphism of drug metabolizing enzymes (DME). Inter-individual variability in drug response can be attributed to polymorphism in genes encoding different DMEs, drug transporters and enzymes involved in DNA biosynthesis and repair [13, 14]. Mutation in gene coding for DMEs may result in variants with high, low or no activity. Major genetic polymorphisms affecting DME activity are related to drug oxidation by cytochrome P450 enzymes (CYP) 2C19, 2C9 and 2D6 [15, 16]. Such polymorphism gives rise to important inter-individual and inter-ethnic variability in the metabolism and disposition of several therapeutic agents resulting in differences in clinical response to these drugs.

The CYP2C19 gene is one of the members of cytochrome P-450 (CYP) super family enzymes involved in metabolism of a number of drugs [15, 17] and accounts for about 2% of oxidative drug metabolism in humans [18]. In the context of this paper, it is important to understand how genetic factors influence CYP2C19 levels and activities. Among the 25 variants of CYP2C19, two principle alleles CYP2C19*2 and CYP2C19*3 have been reported with Poor Metabolizer (PM) phenotype in Caucasians and Asian populations [19, 20]. The presence of the CYP2C19*2 alleles leads to an aberrant splice site, whereas the CYP2C19*3 allele produces a premature stop codon [21]. The importance of this polymorphism has recently been shown as differences in omeprazole concentrations and gastric acid suppression in poor and extensive metabolizers (EMs) of CYP2C19 [22]. Individuals with homozygous (*1/*1) or heterozygous (*1/^∗^2, *1/*3) wild-type CYP2C19*1 genotype have efficient enzyme to metabolize CYP2C19 substrates and are EMs. Individuals with homozygous (*2/*2, *3/*3) or heterozygous mutant CYP2C19*2/CYP2C19*3 genotype have reduced enzyme activity and are termed as PMs.

We chose the CYP2C19 gene polymorphism to study inter-individual variability in drug metabolism and its possible association with metabolically polymorphic *Prakriti*. We investigated the distribution of CYP2C19 genotypes in 132 healthy individuals with different *Prakriti* classes using PCR-RFLP technique. Our study observes an association between CYP2C19 genotype and Ayurveda-based constitution or *Prakriti*.

## 2. Methods

### 2.1. *Prakriti* Evaluation

The *Prakriti* of each subject was assessed using a validated questionnaire based on physical, physiological and psychological characteristics, and clinical judgment of senior Ayurvedic experts. Physique, skin texture, hunger, thirst, digestive capacity, temperament and memory are some of the attributes evaluated to determine individual constitution. The questionnaire also considered information regarding ethnicity, maternal and paternal family history of diseases, past history related to diseases, allergies and dietary habits. Predominant *Prakriti* was allotted if ≥70% dominance of a single Dosha score was obtained. Only individuals with predominance of either of V, P or K were included in the study. Each subject was also assessed clinically by an Ayurvedic expert physician or *Vaidya* who independently classified all subjects into V, P or K groups. Finally, subjects were recruited in this study only when 80% concordance was observed between *Prakriti* assessment with questionnaire scores and clinical evaluation by *Vaidya*. The borderline cases were referred to additional senior *Vaidya* whose decision was considered final.

### 2.2. Subject Screening and Study Population

The study was conducted among the Maharashtrian population residing in Western India and belonging to Australoid-Europoid origin [23]. This avoided any confounding factors due to population stratification. Total 489 subjects were screened and 167 subjects having predominant *Prakriti* types were identified. Subsequently, from these 167 subjects a total of 132 unrelated ethnically matched healthy subjects of both sexes (62 males and 70 females) with predominance of either V (26 subjects), P (43) or K (63) were recruited for the genotyping study, with a mean age of 23.22 (SD 5.3) years. The study protocol was approved by the Ethics Committee of Interdisciplinary School of Health Sciences, University of Pune, India, and written informed consent was obtained from all subjects.

### 2.3. Genotyping for CYP2C19

About 5 mL of venous blood was drawn from each subject in a vacutainer containing EDTA as an anticoagulant. DNA was extracted using Miller's protocol [24]. Genotyping of extracted DNA for CYP2C19*1, CYP2C19*2 and CYP2C19*3 alleles was done using the PCR-RFLP technique as reported earlier [25]. Samples containing mutants were reanalyzed to ensure that the method was 100% reproducible and accurate.

### 2.4. Statistical Analysis

Statistical analysis was performed using the Graph Pad Prism statistical software (San Diego, CA, USA). Using available CYP2C19 genotype data, genotype frequencies were calculated and 2 × 2 contingency tables were constructed using one *Prakriti* group against the remaining two groups. Data related to CYP2C19 genotype and three *Prakriti* types was analyzed using Fisher's exact probability test as the number of individuals in some of the *Prakriti* classes having a particular genotype was very small.

## 3. Results

### 3.1. *Prakriti* Distribution

A total of 132 subjects were classified in three major categories of *Prakriti*: 63 (47.7%) of K, 43 (32.6%) of P and 26 (19.7%) of V.

### 3.2. CYP2C19 Genotype Distribution in *Prakriti* Classes

The genotype frequencies of CYP2C19 polymorphisms in V, P and K *Prakriti* are presented in Table 1. The genotypic repertoire in this population represents predominance of CYP2C19 *1/*2 genotype as compared to other genotypes. This genotype was well distributed in all the three dominant *Prakriti* types. EM genotype (*1/*1, *1/*2, *1/*3) was found to be predominant in P (91%). The *1/*3 genotype specific for the EM group was present only in P. The PM genotype (*2/*2, *2/*3, *3/*3) was highest (31%) in K when compared with V (12%) and P (9%). CYP2C19 *2/*2 genotype frequency was significantly higher with *P* value =  .008 in K type as compared to V + P types yielding an Odds Ratio of 3.5. The *2/*3 genotype typical for PM group was observed only in K *Prakriti.* V *Prakriti* did not show any significant association with any of the genotypes. Although our results did not reach statistical significance, a positive trend was evident in *Prakriti* CYP2C19 genotype association.

## 4. Discussion

Earlier, in an exploratory study correlating HLA DRB1 alleles with ayurvedic *Prakriti* classification, we suggested a genetic basis for the system classification [26]. Present study gives preliminary evidence for the metabolic variability of different *Prakriti* types using DME CYP2C19 gene polymorphism model.

**Figure 1:**
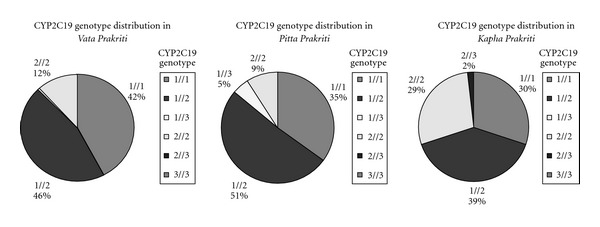


The results obtained in this study suggest possible association of CYP2C19 gene polymorphism with *Prakriti* phenotypes. We observed predominance of EM genotypes (*1/*1, *1/*2, *1/*3) in P *Prakriti* with fast metabolism. The PM genotypes (*2/*2, *2/*3, *3/*3) were highest in K that is expected to be metabolically slow. The CYP2C19 *2/*2 genotype frequency was significantly higher in K type as compared to V + P types. The reported frequencies of *1/*3 and *2/*3 genotypes in Maharashtrian population is very low—1.4% and 0.7%, respectively [25]. It is interesting to note that *1/*3 genotype specific for the EM group was present only in P and *2/*3 genotype typical for PM group was observed in K, as expected. The V type did not show any significant association with any of the genotypes. Some of the genotypes were common to two or more *Prakriti* classes confirming the overlap of *doshas* among *Prakriti* classes (Figure 1). These results allow us to predict that K and P being slow and fast metabolizer groups are likely to require low and high doses of CYP2C19 substrates such as diazepam, certain barbiturates, tricyclic antidepressants, omeprazole and proguanil.

This exploratory study was aimed at investigating metabolic variability in *Prakriti* classes and its relation with the gene polymorphism. One of the limitations of our study is the small sample size and hence, positive associations need to be confirmed with large sample size. Extensive studies on *Prakriti* subtypes and genome wide single nucleotide polymorphism (SNP) mapping especially of other important DME polymorphisms like CYP2D6, CYP2C9, CYP3A4, TPMT, and so forth, would be useful to understand possible *Prakriti* pharmacogenomics relationship correlating genotype, *Prakriti* and drug metabolism. The data generated in this study could be further supported by pharmacokinetic studies on K and P subjects. We have initiated studies on the effect of *Prakriti* and gene polymorphism on drug metabolism and clinical outcome in rheumatoid arthritis patients.

Few earlier studies have attempted to address the relationship between traditional medicine constitutions and HLA polymorphism [27–29]. Studies on whole genome expression [30] and the pharmacogenomics of medicinal plants [31, 32] have also been attempted. There are several similarities between Ayurveda and the Traditional Chinese Medicine (TCM), including the holistic and individual classification systems [33]. Genomics/proteomics correlates of Sasang constitutional medicine (SCM) and Kampo medicine have been reported recently [34–38]. Advancements in the analytical and biological sciences, along with innovations in genomics and proteomics can play an important role in validation of these ancient therapies [39]. Our study demonstrates a probable genomic basis for metabolic differences attributed by *Prakriti.* Identification of genetic variations underlying metabolic variability in Prakriti may provide newer approach to Pharmacogenomics.

## Figures and Tables

**Table 1:** Genotype frequencies of CYP2C19 gene polymorphism in V, P and K *Prakriti*.

CYP2C19 Genotype	Phenotype frequency		*P*	Phenotype frequency		*P*	Phenotype frequency		*P*
	Vata *n* = 26 (%)	Kapha + Pitta *n* = 106 (%)		Pitta *n* = 43 (%)	Kapha + Vata *n* = 89 (%)		Kapha *n* = 63 (%)	Pitta + Vata *n* = 69 (%)	
1/1	11 (42.31)	34 (32.08)	.360	15 (34.88)	30 (33.70)	1.000	19 (30.16)	26 (37.68)	.462
1/2	12 (46.15)	47 (44.34)	1.000	22 (51.16)	37 (41.57)	.351	25 (39.68)	34 (49.27)	.296
1/3	—	2 (1.89)	1.000	2 (4.65)	—	.104	—	2 (2.89)	.497
2/2	3 (11.54)	22 (20.75)	.404	4 (9.32)	21 (23.59)	.059	18 (28.57)	7 (10.14)	**.008**
2/3	—	1 (0.94)	1.000	—	1 (1.12)	1.000	1 (1.59)	—	.477
3/3	—	—	—	—	—	—	—	—	—
